# Classification of optical coherence tomography images using a capsule network

**DOI:** 10.1186/s12886-020-01382-4

**Published:** 2020-03-19

**Authors:** Takumasa Tsuji, Yuta Hirose, Kohei Fujimori, Takuya Hirose, Asuka Oyama, Yusuke Saikawa, Tatsuya Mimura, Kenshiro Shiraishi, Takenori Kobayashi, Atsushi Mizota, Jun’ichi Kotoku

**Affiliations:** 1 Graduate School of Medical and Care Technology, Teikyo University, Tokyo, Japan; 2 Department of Ophthalmology, Teikyo University School of Medicine, Tokyo, Japan; 3 Department of Radiology, Teikyo University School of Medicine, Tokyo, Japan; 4 Central Radiology Division, Teikyo University Hospital, Tokyo, Japan

**Keywords:** Capsule network, Choroidal neovascularization, Deep learning, Diabetic macular edema, Drusen, Optical coherence tomography

## Abstract

**Background:**

Classification of optical coherence tomography (OCT) images can be achieved with high accuracy using classical convolution neural networks (CNN), a commonly used deep learning network for computer-aided diagnosis. Classical CNN has often been criticized for suppressing positional relations in a pooling layer. Therefore, because capsule networks can learn positional information from images, we attempted application of a capsule network to OCT images to overcome that shortcoming. This study is our attempt to improve classification accuracy by replacing CNN with a capsule network.

**Methods:**

From an OCT dataset, we produced a training dataset of 83,484 images and a test dataset of 1000 images. For training, the dataset comprises 37,205 images with choroidal neovascularization (CNV), 11,348 with diabetic macular edema (DME), 8616 with drusen, and 26,315 normal images. The test dataset has 250 images from each category. The proposed model was constructed based on a capsule network for improving classification accuracy. It was trained using the training dataset. Subsequently, the test dataset was used to evaluate the trained model.

**Results:**

Classification of OCT images using our method achieved accuracy of 99.6%, which is 3.2 percentage points higher than that of other methods described in the literature.

**Conclusion:**

The proposed method achieved classification accuracy results equivalent to those reported for other methods for CNV, DME, drusen, and normal images.

## Background

The increase of diabetic patients has come to present difficulty worldwide in recent years. Globally, an estimated 422 million adults were living with diabetes mellitus in 2014, compared to 108 million in 1980 [1]. Diabetes causes diabetic nephropathy, diabetic neuropathy, and diabetic macular edema (DME). In fact, DME might affect up to 746,000 persons with diabetes who are 40 years or older in the United States [2]. Although DME engenders vision loss, early detection and prompt treatment can avert that outcome. From a much broader perspective, age-related macular degeneration (AMD) is expected to affect 8.7% of the worldwide population. The projected number of people with the disease is around 196 million in 2020, increasing to 288 million in 2040 [3]. Early detection and prompt treatment can prevent AMD leading to vision loss. To detect these diseases, optical coherence tomography (OCT) is the most commonly used imaging modality in ophthalmology [4]. These initial diseases can be detected by screening with OCT, but increased screening with OCT images multiplies the burdens on ophthalmologists, who must interpret these images. Therefore, an automatic diagnostic screening system has been developed actively to reduce ophthalmologists’ burdens.

In the field of medical image classification with deep learning [5–10], OCT image classification has been undertaken in earnest. Kermany et al. used Inception-V3 for the classification of OCT images into four classes: choroidal neovascularization (CNV), DME, drusen, and normal. The classification accuracy was reportedly 96.1% [11]. However, traditional convolutional neural networks (CNNs) have sometimes been criticized because their pooling operations nearly eliminate positional information [12]. Losing positional information might be a bottleneck hindering efforts to improve OCT image classification accuracy.

To overcome this shortcoming, Hinton et al. developed a capsule network that can learn positional relations among images using capsules [13–15]. Capsule networks can achieve better performance than existing CNN. For this study using an OCT dataset used for earlier research, we attempted to achieve higher classification accuracy using a model based on a capsule network.

## Methods

### OCT dataset

Kermany et al. released the OCT dataset used for an earlier study to Kaggle [11]. That dataset, which included retinal OCT images, was downloaded from the Kaggle website (https://www.kaggle.com/paultimothymooney/kermany2018, accessed on May 10, 2018). This published dataset includes 84,484 images: 83,484 from the training dataset and 1000 from a test dataset. The dataset included fewer OCT images than the dataset used for the earlier study. The training dataset comprised 37,205 images showing CNV, 11,348 showing DME, 8616 showing drusen, and 26,315 normal images. The test dataset comprised 250 images from each class.

**Fig. 1:**
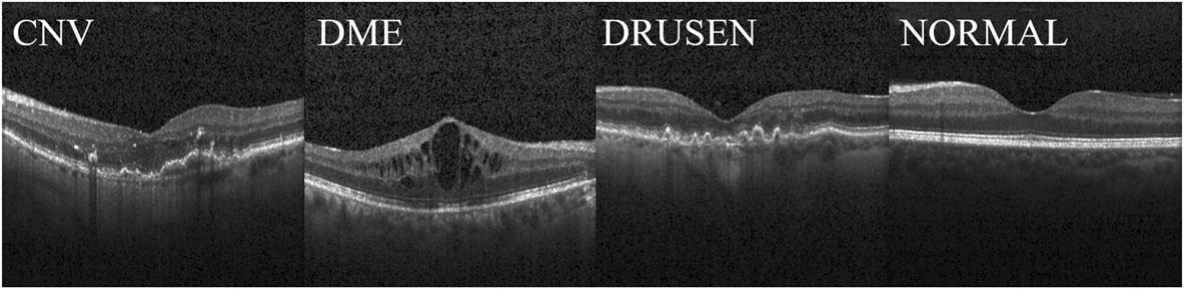
Optical coherence tomography images in the OCT dataset. Panels present images: far left, choroidal neovascularization (CNV); middle left, diabetic macular edema (DME); middle right, drusen; far right, normal

**Fig. 2:**
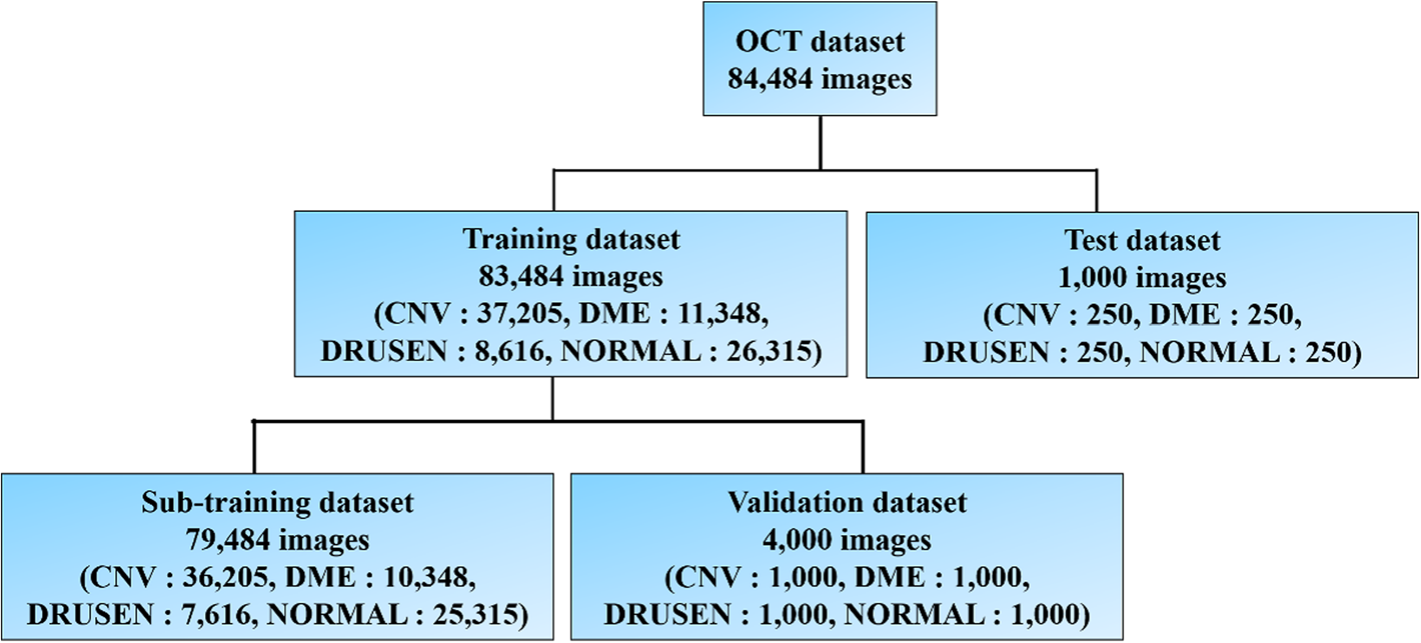
Details of OCT dataset division. The OCT dataset comprises training and test datasets. The training dataset was divided into a sub-training dataset and a validation dataset

We divided the training dataset into a sub-training dataset and a validation dataset, which included 4000 images extracted randomly from 1000 images of each class. The sub-training dataset includes the remaining training data. The image format for the OCT dataset is Joint Photographic Experts Group 8-bit. Figure 1 portrays some OCT dataset images. Figure 2 presents OCT dataset division details.

### Capsule network

Capsules, which are groups of neurons with outputs representing different properties of the same object, have a vector that can learn positional relations between features in images [15]. The coupling coefficients between capsules and all capsules in the layer above it learn with dynamic routing, which enables them to learn positional relations among features. Reportedly, the method provides higher generalization performance than that provided by traditional CNN for small affine transformations of the training data. For that reason, the network requires far fewer training data [13–15].

### Model architecture

#### Capsule network architecture

The original capsule network was a network for classification of MNIST [16] images with 28 × 28 size. The network architecture was shallow, with only two convolutional layers and one fully connected layer. The first layer had 256 filters, 9 × 9 convolution kernels with a stride of 1. The second layer (Primary Caps) was a convolutional capsule layer with 32 channels of convolutional eight dimension (8D) capsules (i.e., each primary capsule contains eight convolutional units with a 9 × 9 kernel and a stride of 2 pixels). Both activation functions were rectified linear units. The final layer (Digit Caps) had one 16D capsule per digit class. Each capsule received input from all capsules of the layer below.

**Fig. 3:**
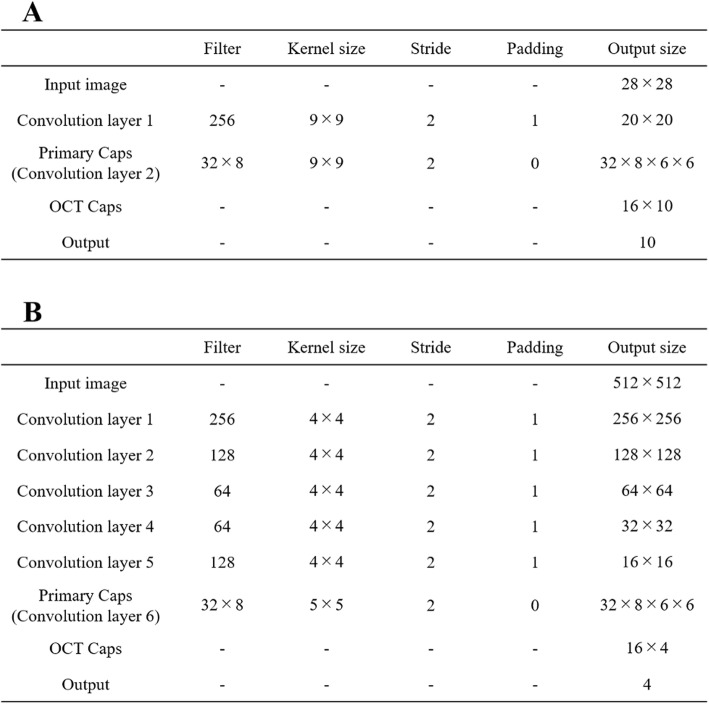
Network architecture details. **a** Capsule network architecture and **b** proposed network architecture

The likelihood vector, elements of which were the likelihood of each digit class, was calculated from the L2 norm of Digit Caps. The output label was the class of the highest component in the likelihood vector [15]. Details of this network architecture are presented in Fig. 3a.

#### Proposed network architecture

**Fig. 4:**
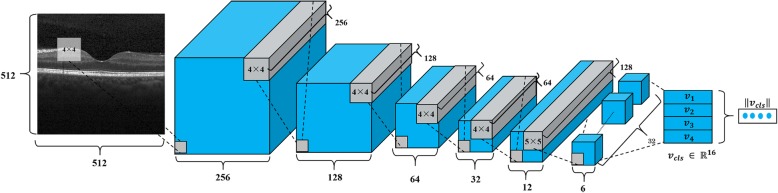
Proposed network architecture. The proposed model has six convolution layers (five convolution layers + primary caps) and OCT Caps. The activity vector length of each capsule in the OCT Caps layer shows the presence of an instance of each class. It is used to calculate the classification loss

For classification of images of 512 × 512, we propose a network model with four added convolutional layers to the capsule network. The first reason is that increasing the convolutional layers of the capsule network was expected to improve accuracy [17]. The second reason is that input images were convolved as the same size as Primary Caps in the capsule network. Figure 4 presents the proposed network architecture. Figure 3b shows some related details.

The first layer has 256 filters: 4 × 4 convolution kernels with a stride of 2 pixels. The second layer has 128 filters. The third and the fourth layers have, respectively, 64 layers. The fifth layer has 128 layers. The sixth layer (Primary Caps) has 32 × 8 filters, 5 × 5 convolutional kernels with a stride of 2 pixels to produce 32 capsule maps with capsules of 8D. This layer constructs capsules for dynamic routing operations in the next layer. The OCT Caps has one 16D capsule per class. Each capsule receives input from all capsules of the layer below. The likelihood vector is calculated from OCT Caps by L2 norm. The highest elements in the likelihood vector are defined as the output label. All activation functions are leaky rectified linear unit (leaky ReLU) functions for which the configurable slope value is 0.05 instead of a rectified linear unit (ReLU) function.

At the Intelligent Systems Research Institute, we built the proposed network model on Reedbush-L running on a computer (Xeon CPUs; Intel Corp. and Tesla P100 16 GB GPU; NVIDIA Corp.) with a Chainer (ver. 3.3.0) deep learning framework.

### Preprocessing and data augmentation

The proposed network model requires a 512 × 512 image. However, the dataset images were 384–1536 pixels wide and 496–512 pixels high. Therefore, the images were resized in terms of width and height to 512 pixels using linear interpolation. In addition, the OCT images were shifted by up to 16 pixels in each direction with zero padding to increase the number of learning data. As a result, the number of images used for learning was increased to 65,536 times (16 × 16 × 16 × 16).

### Learning

The OCT dataset published in Kaggle consists of a training dataset and a test dataset. We trained the proposed model using an early stopping algorithm [18]. Therefore, we divided the training dataset into a validation dataset and sub-training dataset after observing the generalization performance of the proposed model in learning. The validation dataset comprises 4000 images from 1000 images extracted randomly from each class. The sub-training dataset consists of the remaining training dataset. The test dataset had 250 images for each class. The training dataset, the sub-training dataset, the validation dataset, and the test dataset were designated respectively as *X*_train_, *X*_subtrain_, *X*_valid_, and *Y*_test_.

**Fig. 5:**
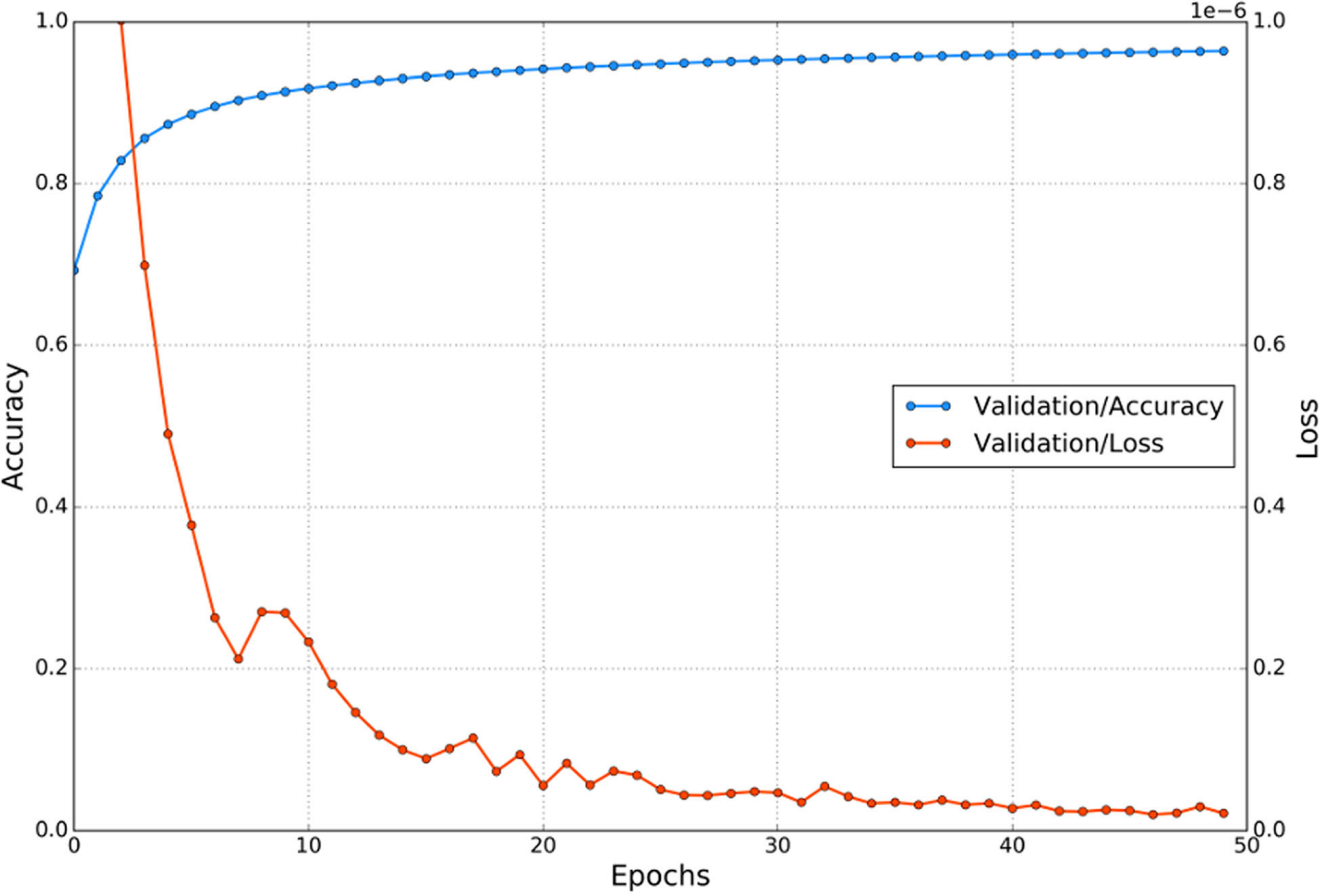
Learning curve. Early stopping occurred when the validation dataset accuracy was the best in learning

The model was trained with *X*_subtrain_ and *X*_valid_ using Adam optimizer [19]. The batch size was set to 128. The model was trained for 50 epochs. Early stopping occurred when the *X*_valid_ accuracy became the best in learning. This learning curve is depicted in Fig. 5. Then, the proposed model was evaluated using the test dataset.

Additionally, we trained Inception-v3 under the same learning conditions to compare the proposed model and those of earlier research. Then, Inception-V3, which was trained, was evaluated using the test dataset.

### Visualizing feature maps

We visualized feature maps using a method inspired by class activation mapping (CAM) [20] to elucidate which parts in the OCT image were strongly influential. An image was input to the trained model. Then 256 feature maps (6 × 6) were generated from Convolution layer 6. After the averaged feature map (6 × 6) was resized to input size (512 × 512), it was superimposed on the input image as a heat map image.

## Results

**Fig. 6:**
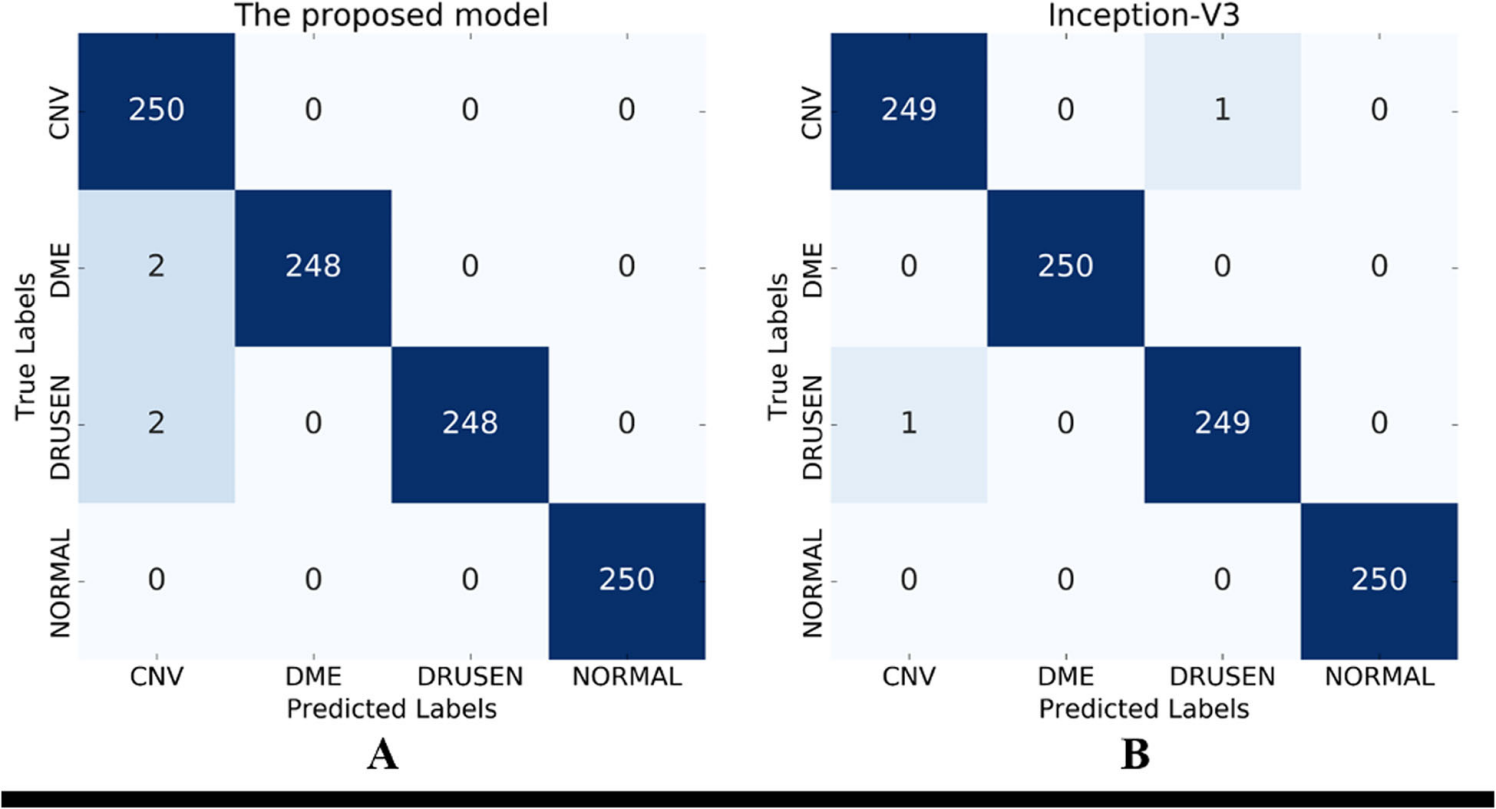
Confusion matrixes of learned model classification. **a** Confusion matrix by the proposed model and **b** used by Inception-V3

We evaluated the proposed model using the *Y*_test_ test dataset. The numbers of correct answers and rates of CNV, DME, drusen, and normal were, respectively, 250 (100%), 248 (99.2%), 248 (99.2%), and 250 (100%). The model achieved average classification accuracy of 99.6%. More detailed results are presented as a confusion matrix in Fig. 6a.

We evaluated Inception-V3 similarly. The accuracy of Inception-V3 is presente in Fig. 6b as a confusion matrix. The numbers of correct answers and rates of CNV, DME, drusen, and normal were, respectively, 249 (99.6%), 250 (100%), 249 (99.6%), and 250 (100%). Consequently, the average accuracy achieved using Inception-V3 was 99.8%.

**Fig. 7:**
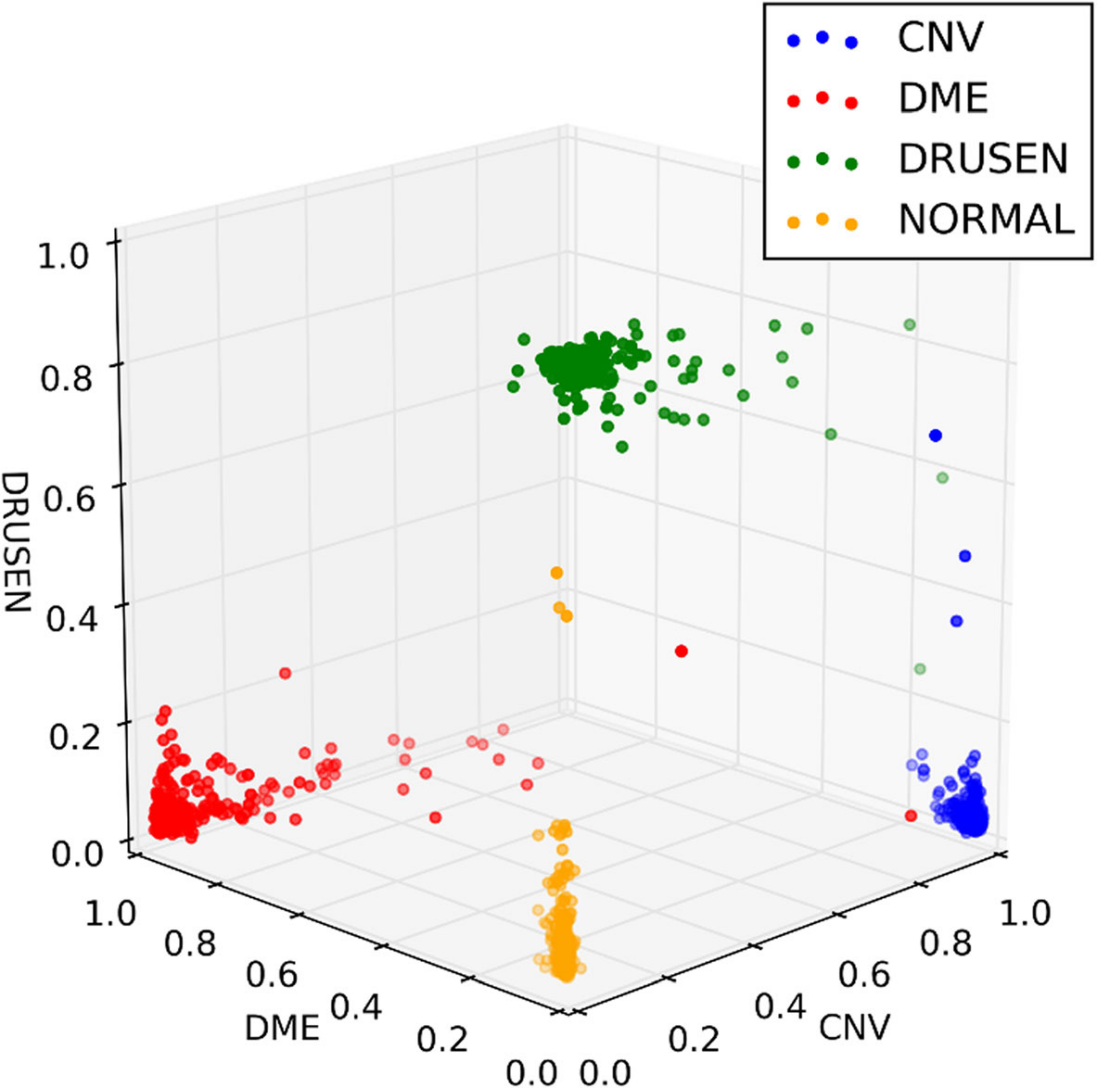
Visualizing of likelihood vectors in the four classes. Likelihood vectors calculated from the test dataset are shown. Axes show the likelihood of diseases of three kinds. Blue, red, green, and yellow lines respectively represent true labels of CNV, DME, DRUSEN, and NORMAL

We visualized likelihood vectors, for which coefficients denote the probability of each class, as calculated from the OCT Caps. Components of likelihood vectors are presented in Fig. 7, the axes of which respectively show the likelihoods of CNV, DME, and DRUSEN. The marker colors correspond to the correct labels of four classes in the test dataset. Also, CNV, DME, DRUSEN, and NORMAL are presented respectively as blue, red, green, and yellow plotted values.

**Fig. 8:**
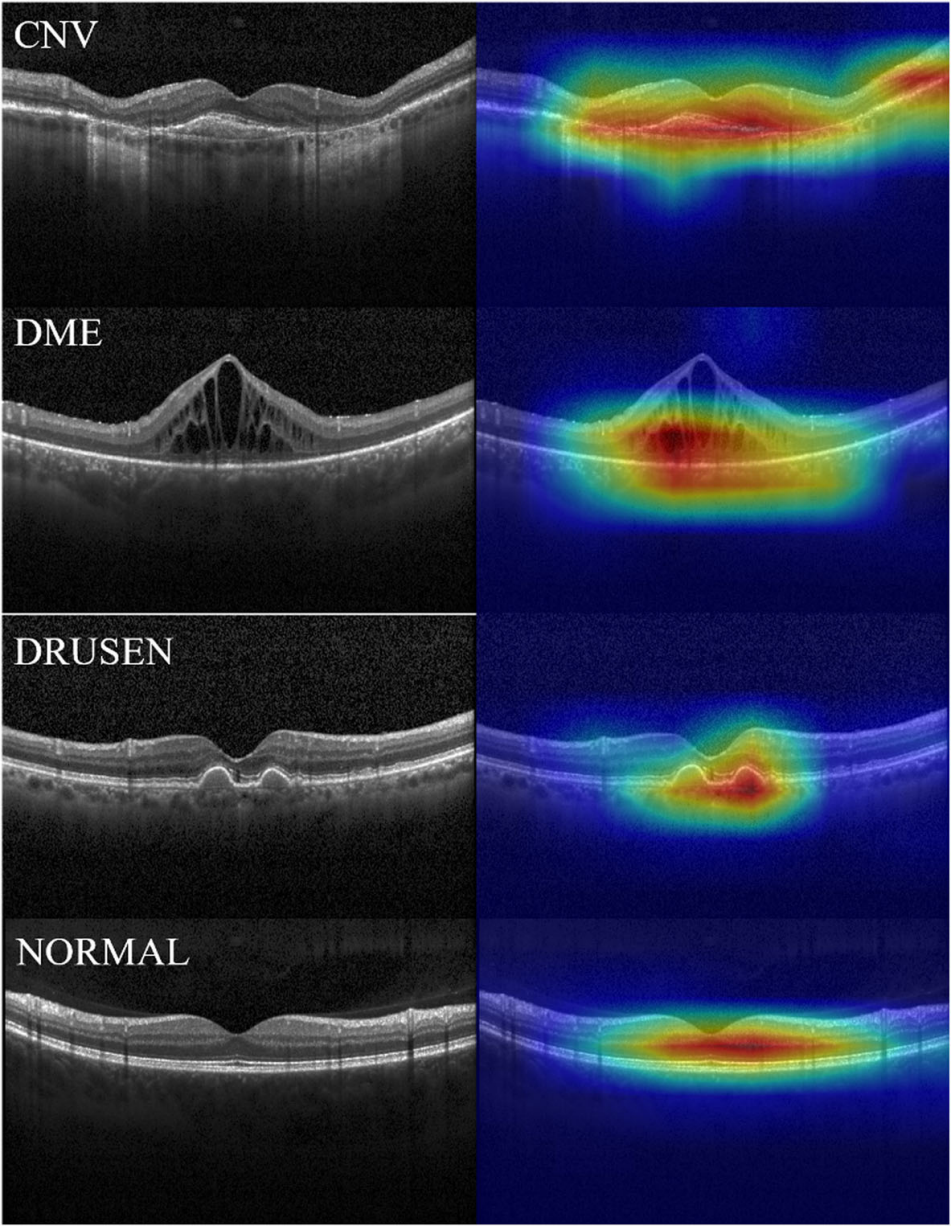
Visualization of feature maps as heat maps. Left images are input images. Right images are feature maps superimposed on an input image. Top to bottom, panels show CNV, DME, DRUSEN, and NORMAL

**Fig. 9:**
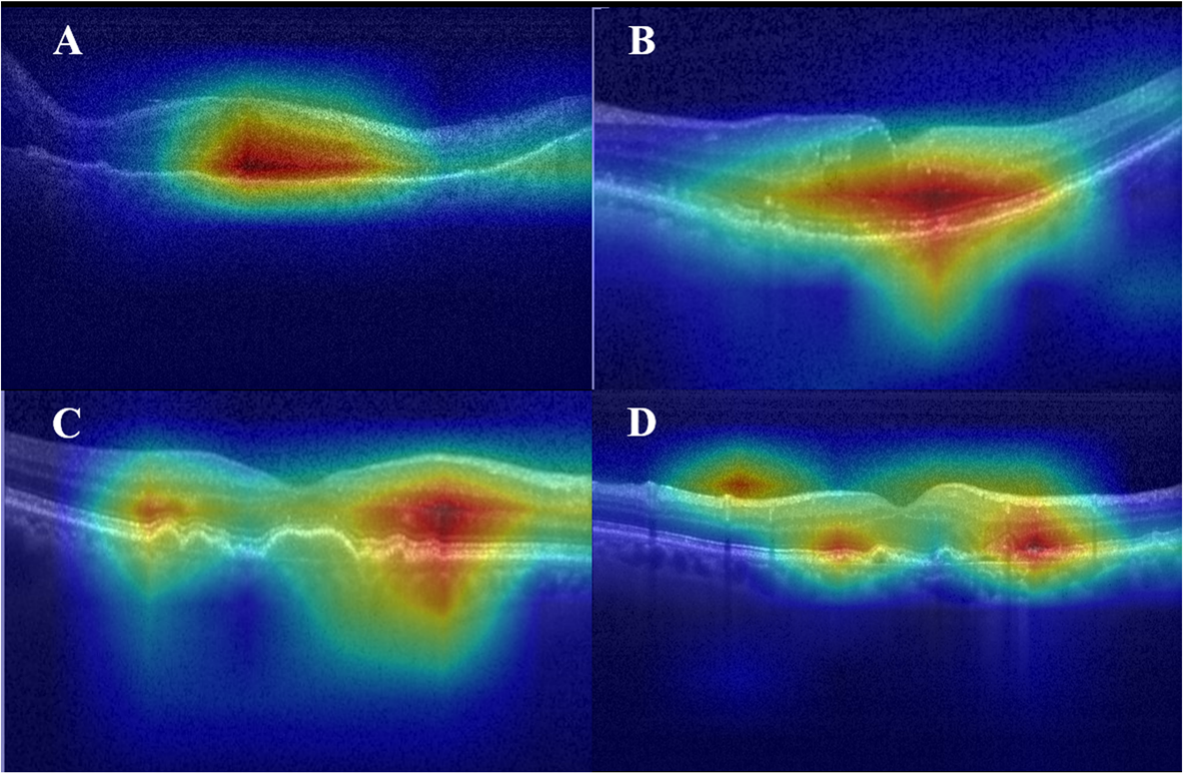
Visualization of feature maps misclassified by the proposed model. The proposed model misclassified DME (**a** and **b**) and DRUSEN (**c** and **d**) as CNV

Heat map images for the respective classes are portrayed in Fig. 8. The red zones in heat map images show activated parts of the proposed model. After expert ophthalmologists assessed the heat map images, they confirmed that the activated parts corresponded to the observed location in the interpretation of OCT image. Therefore, it can be said, at least qualitatively, that the proposed model was trained accurately. Additionally, heat map portrayals of images that were misclassified by the proposed model are presented in Fig. 9.

## Discussion

For the classification of four classes of OCT images, the proposed method achieved high accuracy of 99.6% using the network model based on a capsule network. By contrast, the best accuracy obtained in earlier studies was 96.1%, obtained when using Inception-v3. This model has a pooling layer, which is a primary feature of CNN. In addition, the accuracy of Inception-V3 in the same condition was 99.8%. Therefore, the proposed model, which is much shallower than Inception-V3, compares favorably with it in terms of classification accuracy.

According to an earlier study [21], six ophthalmologists diagnosed the same test dataset and achieved classification accuracies of 92.1–99.7%, with mean accuracy of 96.7%. That finding suggests that the proposed network model performance in terms of OCT image classification was equivalent to those of expert ophthalmologists.

Misclassified heat map images suggest that the activated parts in those images are the same locations that ophthalmologists interpret. Therefore, the proposed model did not specifically examine wrong parts. One can infer that those misclassified images include some disease.

In a typical clinical case, a fundus image is taken using fluorescein angiography in addition to OCT images. Fluorescein angiography nevertheless presents several important shortcomings. The first is a contraindication to perform fluorescein angiography for patients with severe cardiac disease, severe cerebrovascular disease, severe diabetes, and liver cirrhosis [22–24]. Moreover, it is impossible to obtain contrast agents for pregnant women [25, 26]. The second shortcoming is the risk of side effects presented by fluorescein angiography, such as anaphylaxis (difficulty of breathing and loss of consciousness) and cardiac arrest [24, 27, 28]. For this study, the proposed model enables the classification of CNV, DME, drusen, and normal with high accuracy using OCT images alone. Therefore, the proposed model can reduce burdens imposed on ophthalmologists and patients.

An important limitation of this study is that the proposed model classifies images of only four types: CNV, DME, drusen, and normal. Retinal disease, such as glaucoma, Branchi Retinal Vein Occlusion cannot be predicted because these diseases were not trained in this study. Future studies will be conducted to classify those images using this method.

## Conclusions

This network model with four convolution layers of an added capsule network achieved high accuracy for the released OCT dataset. Results obtained for the four classifications compare favorably with those reported from earlier research. This system can reduce ophthalmologists’ burdens and can be expected to improve patient access to rapid treatment.

## Data Availability

All data analyzed during this study are available from Daniel Kermany, Kang Zhang, and Michael Goldbaum (2018), “Labeled Optical Coherence Tomography (OCT) and Chest X-Ray Images for Classification”, Mendeley Data v2 (10.17632/rscbjbr9sj.2). Data generated from analyses described in the *Results* section are deposited from the corresponding author upon request.

## Abbreviations

CNN: Convolutional neural network
OCT: Optical coherence tomography
CNV: Choroidal neovascularization
DME: Diabetic macular edema
AMD: Age-related macular degeneration
ReLU: Rectified linear unit
CAM: Class activation mapping
